# Kinetic study of hydroxyl radical formation in a continuous hydroxyl generation system

**DOI:** 10.1039/c8ra08511k

**Published:** 2018-12-05

**Authors:** Xin Wang, Long Zhang

**Affiliations:** Jilin Provincial Engineering Laboratory for the Complex Utilization of Petro-resources and Biomass, School of Chemical Engineering, Changchun University of Technology Changchun Jilin 130012 P. R. China zhanglongzhl@163.com +8618686672766; School of Petrochemical Technology, Jilin Institute of Chemical Technology Jilin 132022 P. R. China

## Abstract

A novel and simple apparatus for the continuous generation of hydroxyl radicals has been constructed for the first time. In this paper, we focused on the investigation into the kinetic study of hydroxyl radical formation in the preparation process. The effects of the process parameters (such as the electrolyte solution concentration, graphite dosage, the applied current strength, and air flow rate) on the concentration of hydroxyl radicals were investigated in detail. The concentration of hydroxyl radicals first increased with the concentration of sodium dodecyl benzene sulfonate electrolyte solution, graphite dosage, applied current strength, and air flow rate, and then decreased. The concentration of ·OH and time well fit a third-order model of {C(·OH) = *B*_1_ × *t* + *B*_2_ × *t*^2^ + *B*_3_ × *t*^3^ + intercept}. The highest concentration of hydroxyl radicals was 7.98 × 10^−3^ mol L^−1^ under the following conditions: sodium dodecyl benzene sulfonate concentration 10.0% (w/v), graphite dosage 5.0 g, applied current strength 10 mA, and air flow rate 1.0 L h^−1^. Our hydroxyl radical generation method can achieve the preparation of higher-concentration hydroxyl radicals continuously without using strong acid reagents. Moreover, our method has low energy consumption by using milliampere-level current. It is a green and efficient method for the generation of hydroxyl radicals. The kinetic study of hydroxyl radical generation can quantitatively predict the concentration changes with process parameters and provide a good prediction of hydroxyl radical generation, which is crucially important in industrial applications.

## Abstract 2

A novel and simple apparatus for the continuous generation of hydroxyl radicals has been constructed for the first time.

## Graphical abstract

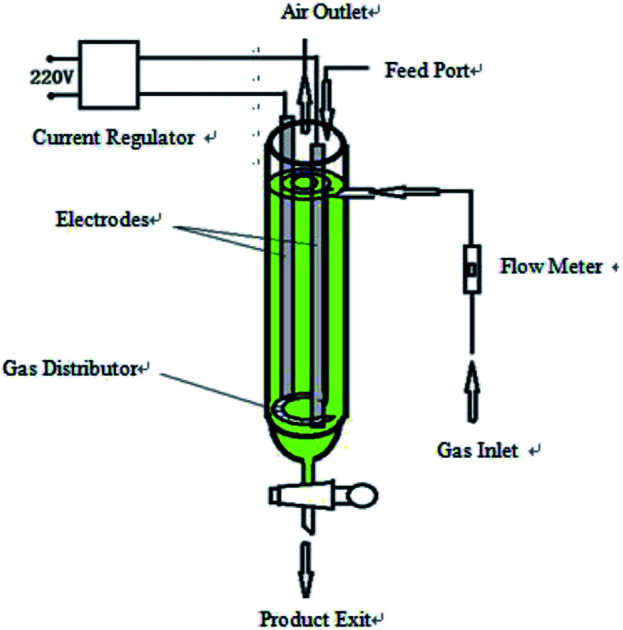

## 1. Introduction

The hydroxyl radical (·OH) has a standard oxidation–reduction potential of 2.8 V, which is only lower than that of F.^1^ As its oxidation characteristics are non-selective and it can react with almost all types of substances, ·OH can initiate and perform many free radical oxidation reactions. The hydroxyl radical mainly oxidizes soluble inorganic and organic substances through electron transfer, dehydrogenation, addition and self-quenching.^2^ The hydroxyl radical is mainly used in the field of industrial sewage treatment,^3–7^ sterilization and preservation,^8–10^ carbon nano-tube modification^11^ and zeolite preparation.^12^ Hydroxyl radicals have low concentrations in the above applications, and their existence depends on specific complex conditions. In the current reports,^13,14^ the concentration of hydroxyl radicals prepared by the common preparation methods are about 10^−4^ mol L^−1^, and the preparation process is complicated. At present, the common methods to generate hydroxyl include Fenton reaction,^8,9^ Haber–Weiss reaction^8^ and electrochemical methods.^9,10,15^ These hydroxyl radical generation methods have the disadvantages of high energy consumption, intermittent generation, complex production procedures and hence, they not suitable for mass applications. Our method of generating hydroxyl radical can continuously generate hydroxyl radical without using strong acid reagents, so that it will not pollute the environment. At the same time, our method involves low energy consumption by using milliampere-level current. In summary, our method is a green and efficient method for the generation of hydroxyl radical.

In our previous research, we designed and set up a continuous hydroxyl radical generation apparatus for the first time, and achieved the facile exfoliation of graphite to prepare graphene.^16^ For industrial applications and mass production, the kinetic study of hydroxyl radical generation is necessary, because it can give a good prediction for hydroxyl radical generation kinetics in experiments, which allows us to estimate the initial rate and extent of our continuous and efficient hydroxyl radical generation method. Research on the kinetics of our continuous hydroxyl radical generation method has not yet been reported. Herein, we used a third-order model to study the kinetics mechanism of our method, in order to establish the generation kinetic equation. Furthermore, we studied the influences of the sodium dodecyl benzene sulfonate (SDBS) solution concentration, graphite dosage, applied current strength, and air flow rate on the concentration changes of hydroxyl radicals.

## 2. Experimental

### 2.1 Materials and instruments

Flake graphite (0.5 mm) was purchased from Sinopharm Chemical reagent Co. (Shanghai, China). Sodium dodecyl benzene sulfonate (SDBS) (AR), sulfuric acid (AR), ferrous sulfate (AR), potassium dichromate (AR), and ethanol (AR) were purchased from Sinopharm Chemical reagent Co. (Shanghai, China).

**Fig. 1:**
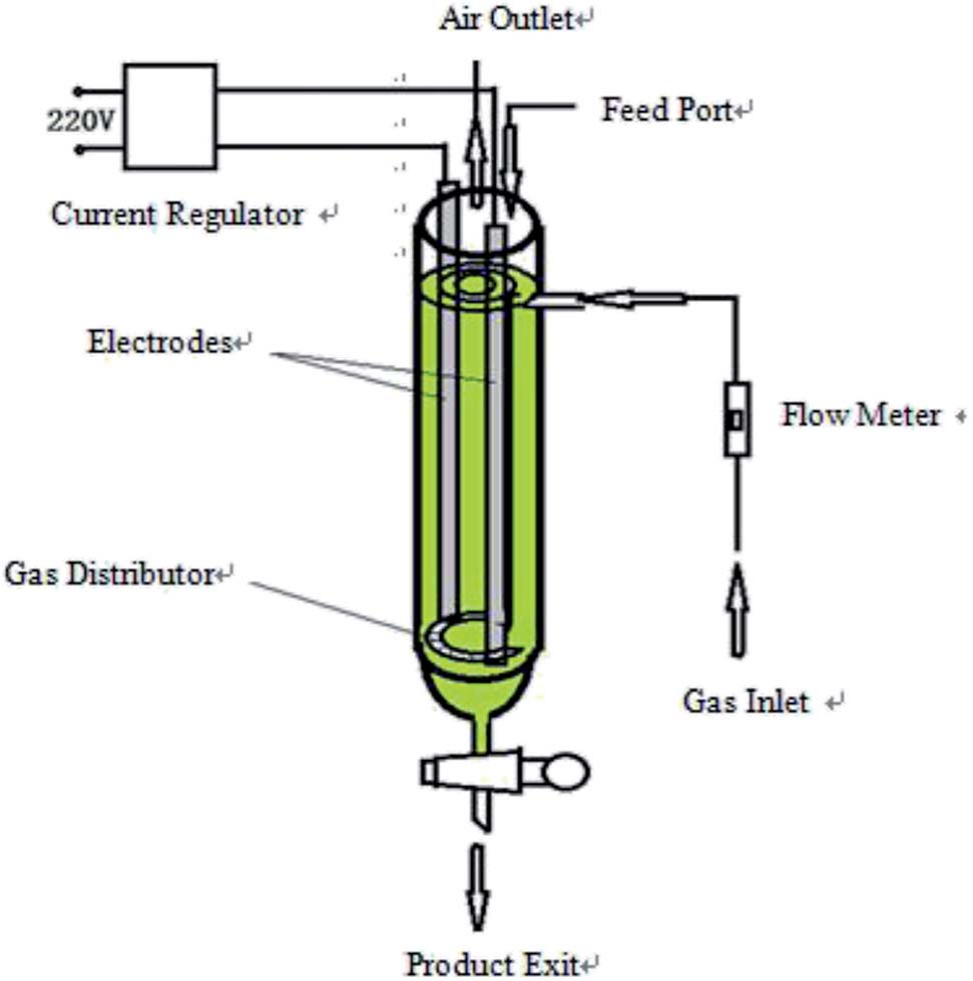
The diagram of hydroxyl radical generation apparatus.

The hydroxyl radical production apparatus was designed and manufactured by our laboratory; the detailed device diagram is shown in Fig. 1. The automatic potentiometric titrator (ZDJ-4A) was purchased from Shanghai Yidian Scientific Instrument Co. (Shanghai, China).

### 2.2 Experimental devices

The device shown in Fig. 1 is a high efficiency hydroxyl radical generation apparatus designed by our laboratory.

The apparatus consists of a current regulator, electrode system, gas inlet and distributor, flow meter, feed port, air outlet and product exit. Inside the device casing is a draft tube, and the gas distributor is at the bottom of the draft tube to connect the outer casing and the draft tube. The two electrodes are distributed among the outer casing and the draft tube and are arranged symmetrically. This method uses a certain concentration of electrolyte as reaction liquid and graphite as catalyst to generate hydroxyl radicals. The reaction liquid and graphite were added from the feed port, and the liquid level was higher than the upper edge of the electrodes and the inner guide tube. The air flow rate was adjusted by a flow meter. Graphite was evenly distributed among the reaction liquid under the effect of an air distributor. The liquid in the outer casing forms an internal circulation system between the draft tube and the outer casing under the effect of the airflow, and the catalyst is in contact with the reaction liquid in the circulating flow of the liquid. The intensity of the applied current is adjusted by the current regulator. When an electric current was applied, water was decomposed and oxidized to produce ·OH in the reactor with graphite as a catalyst, which subsequently continuously generated hydroxyl radicals.

### 2.3 Kinetic experiments of hydroxyl radical formation

Graphite powders were loaded into our self-designed hydroxyl radical production apparatus containing a volume of electrolyte solution and flake graphite serving as the catalyst, according to the experimental design. The applied current strength in the process was adjusted by a variable resistor having 50–500 Ω. The kinetic experiments were started when the electric current was applied. The taken sample volume was 2.0 mL each time. The concentration of hydroxyl radicals in the sample was measured by the titration method. The samples were taken at 10, 20, 40, 60, 80, 100, 120, 150 and 180 min intervals.

### 2.4 Analytic method and calculation

At present, the main detection methods are chemical fluorescence method,^17^ fluorescence,^18^ electron spin capture,^19–23^ chromatography,^24^ and so on.^25–28^ Some of these methods require expensive instruments, and some operations are very complicated. In this paper, the combined electrochemical and redox method was employed to measure the hydroxyl radical concentration, making the measurement of concentration of hydroxyl radicals in the kinetic experiments simple and efficient.

Concentration of ·OH was determined by redox titration in an automatic potentiometric titrator. The platinum electrodes were used as the indicator electrode and the saturated calomel electrode was used as the reference electrode. The calculation principle is as follows: excess Fe^2+^ is reacted with hydroxyl radical and then, the remaining Fe^2+^ is back titrated with potassium dichromate. The main chemical reaction equations in the titration process are as follows:

1 H^+^ + Fe^2+^ + ·OH → Fe^3+^ + H_2_O

2 Cr_2_O^2−^_7_ + 6Fe^2+^ + 14H^+^ → 6Fe^3+^ + 2Cr^3+^ + 7H_2_O

First, 20 mL of 0.05 mol L^−1^ ferrous sulfate solution was added to a conical flask (1#, 2#). Following this, 20 mL of sample solution was added to the 1# conical flask along with 10 mL of 1 mol L^−1^ sulfuric acid. The remaining Fe^2+^ was back titrated with a known concentration of potassium dichromate solution to an automatic potentiometric titrator. Upon addition of 10 mL of 1 mol L^−1^ sulfuric acid to the 2# conical flask, the potentiometric titration was performed directly with potassium dichromate solution. The hydroxyl radical concentration {C(·OH)} can be calculated from the difference in the amount of potassium dichromate used in the potentiometric titration between the blank and the sample {Δ*V*(K_2_Cr_2_O_7_)}. The calculation formula is as follows (3):

3 C(·OH) = [6 × *C*(K_2_Cr_2_O_7_) × Δ*V*(K_2_Cr_2_O_7_)]/*V*(·OH)

*C*(K_2_Cr_2_O_7_) – concentration of potassium dichromate (mol L^−1^), *V*(·OH) – volume of sample containing hydroxyl radicals (L).

### 2.5 Kinetic data regression

Using Origin 8.0 to fit the data, the concentration of ·OH and times were fitted by the third-order model as follows (4):

4 C(·OH) = *B*_1_ × *t* + *B*_2_ × *t*^2^ + *B*_3_ × *t*^3^ + intercept

*C*(·OH) – concentration of hydroxyl radicals formed (mol L^−1^), *t* – sampling time (min), *B*_1_, *B*_2_, *B*_3_, intercept – constant fitted by software.

## 3. Results and discussion

### 3.1 Electrolyte SDBS solution concentration

**Fig. 2:**
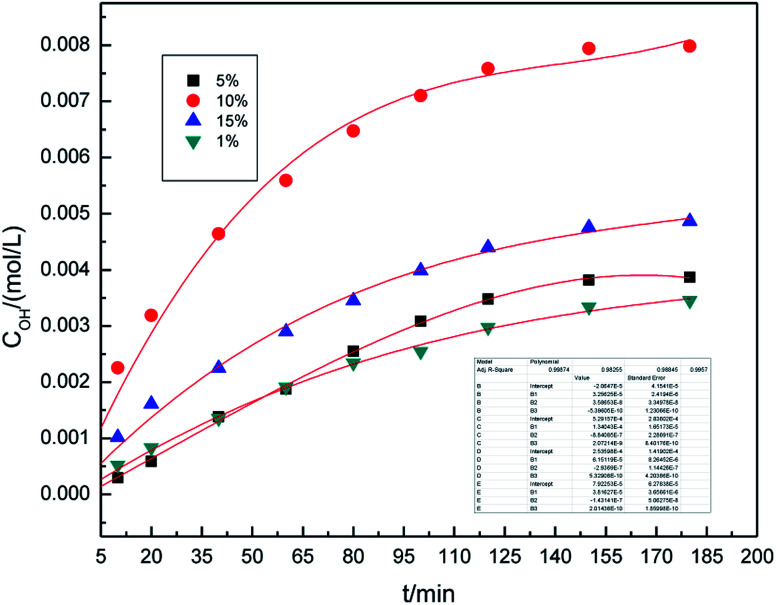
Effect of SDBS concentration on the concentration of hydroxyl radicals.

In our previous study,^16^ we investigated the roles of different electrolyte systems on the generation of hydroxyl radicals, and found that the SDBS demonstrated the greatest effect. First, to study the effect of SDBS concentration on the generation of hydroxyl radicals in the preparation process, the SDBS concentration was varied from 1.0% (w/v) to 10.0% (w/v), and the samples were analyzed at 10, 20, 40, 60, 80, 100, 120, 150 and 180 min. The other parameters of preparation process were set as follows: the air flow rate was 1.0 L h^−1^, the applied current strength was 10 mA, and the dosage of graphite powder was 3.0 g. The results are shown in Fig. 2.

It can be easily seen from Fig. 2 that the concentration of hydroxyl radical increases significantly overtime, and remains essentially unchanged after 160 minutes. On fitting the data with Origin 8.0, we found that a third-order model appropriately describes the changes of hydroxyl radical concentration overtime. For all concentrations, the *R*^2^ are 0.993 and the standard error is below 0.02, indicating that the third-order model, as described by formula (4), is well applicable.

The concentration of hydroxyl radicals increased with the increase in electrolyte concentration, reaching the maximum at 10% (w/v), and then decreased. This phenomenon can be attributed to an increase in the conductivity of the solution when higher concentration of electrolyte is used. Good electrical conductivity is favorable for ·OH generation. Furthermore, the concentration of hydroxyl radicals decreased due to less water in the higher electrolyte concentration. The optimal electrolyte concentration for the generation of hydroxyl radicals in different electrolyte systems by different production methods were also reported in the literature.^29–31^ Li *et al.*^31^ used a high-efficiency power reactor to generate hydroxyl radicals and showed that the optimal electrolyte concentrations were 3.0–6.0% (w/v). Therefore, it is reasonable to expect that an optimal concentration of electrolyte may exist. As can be inferred from the results, 10.0% (w/v) SDBS concentration was found to be optimal for the generation of hydroxyl radicals. Thus, 10.0% (w/v) SDBS concentration was adopted in the subsequent experimental runs.

### 3.2 Applied current strength

**Fig. 3:**
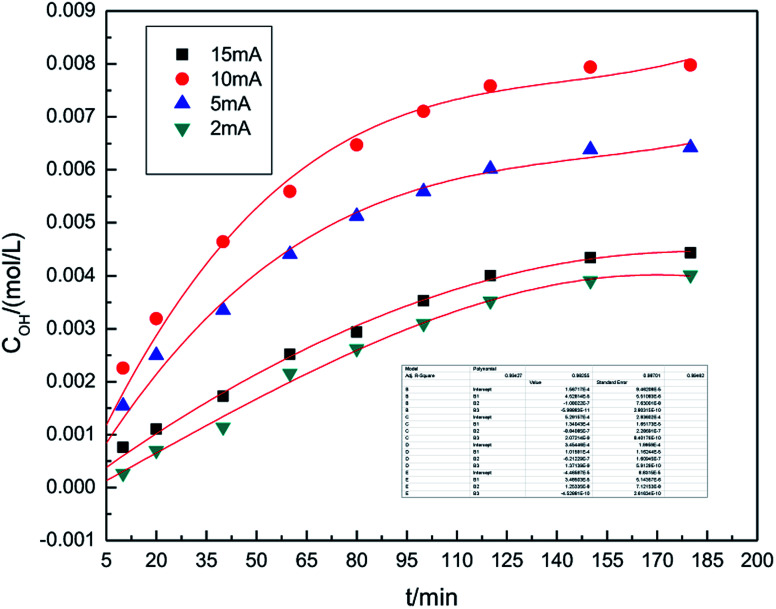
Effect of applied current strength on the concentration of hydroxyl radicals.

The experiments studying the effect of applied current strength on the generation of hydroxyl radicals was performed at the air flow rate of 1.0 L h^−1^, and the dosage of graphite powder was 3.0 g. The applied current strength ranged between 2 mA and 15 mA. The results are shown in Fig. 3. This indicates that when the applied current intensity increases, the concentration of hydroxyl radicals first increases, achieves a maximum at 10 mA and then decreases. Within a certain range, a relatively high applied current intensity indicates higher power input, which can result in higher hydroxyl radical generation. However, upon further increasing the current, the cathode and anode will generate hydrogen and oxygen, respectively, as side products.^32,33^ The reactions are shown as follows ((5) and (6)). Bipolar side effect leads to the decrease in hydroxyl radical generation.

5 2H_2_O − 4e → O_2_↑ + 4H^+^

6 2H^+^ + 2e → H_2_↑

To ensure a higher concentration of hydroxyl radicals, 10 mA was chosen as the optimal applied current strength.

### 3.3 Graphite dosage

**Fig. 4:**
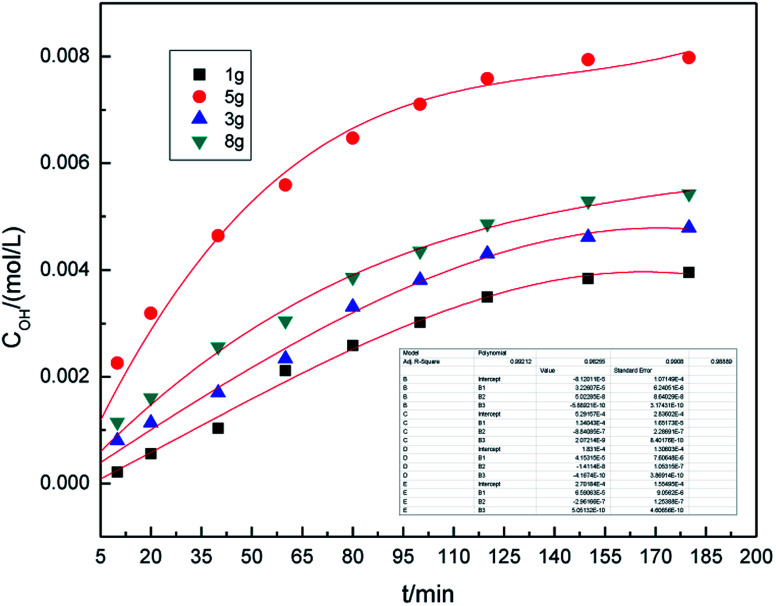
Effect of graphite dosage on the concentration of hydroxyl radicals.

The dosage of graphite is a key parameter because graphite shows catalytic activity towards the generation of hydroxyl radicals. Fig. 4 shows the effect of the graphite dosage on the generation of hydroxyl radicals at the air flow rate of 1.0 L h^−1^ and the dosage of graphite varied from 1.0 g to 8.0 g.

As can be seen from Fig. 4, as the amount of graphite increases, the concentration of hydroxyl radicals increases first, reaches the maximum at 5.0 g, and then decreases. This is because higher dosage of graphite results in better catalytic activity, which leads to an increase in the generation of hydroxyl radicals. At the same time, the higher the dosage of graphite led to the graphite dispersing unevenly, leading to a decrease in the catalytic activity. This reduced the concentration of hydroxyl radicals. From these results, we inferred that a suitable dosage of graphite was 5.0 g.

### 3.4 Air flow rate

**Fig. 5:**
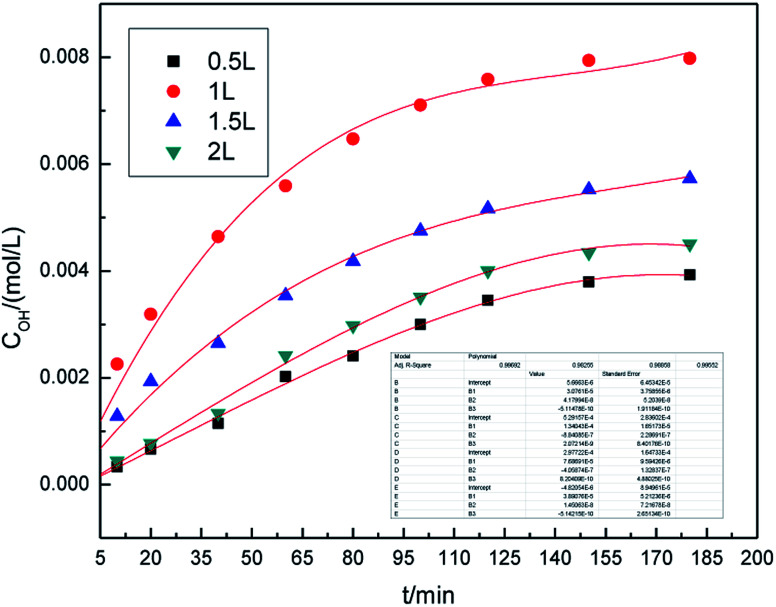
Effect of air flow rate on the concentration of hydroxyl radicals.

In general, higher air flow rate allows for better contact of graphite with the electrolytes, thus improving the generation of hydroxyl radicals. From an economic point of view, the use of high air flow rate is not considered as cost effective due to high operating costs. The effect of air flow rate on the generation of hydroxyl radicals is shown in Fig. 5. The air flow rate ranged between 0.5 L h^−1^ and 2.0 L h^−1^. From Fig. 5, it can be seen that the concentration of hydroxyl radicals first increased with the increase in air flow rate, reached to the maximum at 1.0 L h^−1^, and then decreased. This is because higher air flow rate can accelerate the mass transfer and favor the generation of ·OH. When the air flow rate was too high, the mass transfer interface area and contact time decreased, which was not conducive to ·OH generation and resulted in a decrease in the concentration of hydroxyl radicals. Therefore, the flow rate of 1.0 L h^−1^ was chosen.

From the kinetic study of hydroxyl radical generation, we found that the concentration of hydroxyl radical first increased with the concentration of SDBS solution, graphite dosage, applied current strength, and air flow rate, and then decreased. The optimal generation conditions of hydroxyl radicals were as follows: SDBS electrolyte concentration, 10.0% (w/v); graphite dosage, 5.0 g; applied current strength, 10 mA; and air flow rate, 1.0 L h^−1^. At these conditions, the highest concentration of hydroxyl radicals was 7.98 × 10^−3^ mol L^−1^.

## 4. Conclusion

In this study, we investigated a new method to generate hydroxyl radicals simply and continuously. In order to study the formation kinetics of hydroxyl radical generation, we studied the influences of the SDBS solution concentration, graphite dosage, applied current strength, and air flow rate on the concentration of hydroxyl radicals. Results showed that the concentration of hydroxyl radical first increased with the concentration of SDBS solution, graphite dosage, applied current strength, and air flow rate, and then decreased. The third-order model, C(·OH) = *B*_1_ × *t* + *B*_2_ × *t*^2^ + *B*_3_ × *t*^3^ + intercept, well describes the relationship of hydroxyl radical concentration and time. The optimal generation conditions of hydroxyl radicals were as follows: SDBS electrolyte concentration 10.0% (w/v), graphite dosage 5.0 g, applied current strength 10 mA, and air flow rate 1.0 L h^−1^. Under these conditions, the concentration of hydroxyl radicals was 7.98 × 10^−3^ mol L^−1^. Our hydroxyl radical generation method can achieve the preparation of high concentration hydroxyl radicals continuously without using strong acid reagents. Moreover, our method involves low energy consumption since only milliampere-level current was used. This is a green and efficient method for the generation of hydroxyl radicals. The kinetic study of hydroxyl radical generation can quantitatively predict the concentration changes with process parameters and allow us to estimate hydroxyl radical concentration and the process parameters control.

## Conflicts of interest

There are no conflicts to declare.

## Supplementary Material
